# Explainable machine learning radiomics model for Primary Progressive Aphasia classification

**DOI:** 10.3389/fnsys.2024.1324437

**Published:** 2024-03-18

**Authors:** Benedetta Tafuri, Roberto De Blasi, Salvatore Nigro, Giancarlo Logroscino

**Affiliations:** ^1^Department of Translational Biomedicine and Neuroscience (DiBraiN), University of Bari Aldo Moro, Bari, Italy; ^2^Center for Neurodegenerative Diseases and the Aging Brain, University of Bari Aldo Moro at Pia Fondazione “Card. G. Panico”, Tricase, Italy

**Keywords:** Primary Progressive Aphasia, MRI, machine learning (ML), radiomics, explainability

## Abstract

**Introduction:**

Primary Progressive Aphasia (PPA) is a neurodegenerative disease characterized by linguistic impairment. The two main clinical subtypes are semantic (svPPA) and non-fluent/agrammatic (nfvPPA) variants. Diagnosing and classifying PPA patients represents a complex challenge that requires the integration of multimodal information, including clinical, biological, and radiological features. Structural neuroimaging can play a crucial role in aiding the differential diagnosis of PPA and constructing diagnostic support systems.

**Methods:**

In this study, we conducted a white matter texture analysis on T1-weighted images, including 56 patients with PPA (31 svPPA and 25 nfvPPA), and 53 age- and sex-matched controls. We trained a tree-based algorithm over combined clinical/radiomics measures and used Shapley Additive Explanations (SHAP) model to extract the greater impactful measures in distinguishing svPPA and nfvPPA patients from controls and each other.

**Results:**

Radiomics-integrated classification models demonstrated an accuracy of 95% in distinguishing svPPA patients from controls and of 93.7% in distinguishing svPPA from nfvPPA. An accuracy of 93.7% was observed in differentiating nfvPPA patients from controls. Moreover, Shapley values showed the strong involvement of the white matter near left entorhinal cortex in patients classification models.

**Discussion:**

Our study provides new evidence for the usefulness of radiomics features in classifying patients with svPPA and nfvPPA, demonstrating the effectiveness of an explainable machine learning approach in extracting the most impactful features for assessing PPA.

## 1 Introduction

Primary Progressive Aphasia (PPA) is a neurodegenerative disorder that affects approximately three to four individuals per 100,000 (Coyle-Gilchrist et al., 2016). PPA is the second major form of Frontotemporal Lobe Degeneration (FTLD) and is clinically characterized by language deficits affecting speaking, writing and comprehension skills (Mesulam, 1982; Gorno-Tempini et al., 2011; Leyton et al., 2011; Tee and Gorno-Tempini, 2019). The two most distinctive subtypes of PPA include non-fluent/agrammatic variant (nfvPPA), characterized by slow, labored speech and grammatical errors, and semantic variant (svPPA), marked by an inability to comprehend words or construct sentences. Each variant exhibits specific phenotypic features corresponding to the underlying pathology. SvPPA is typically linked to TDP-43-C pathological aggregates (75–100% of patients) and also most often associated with FTD tau pathology (Spinelli et al., 2017). On the contrary, nfvPPA is commonly related to a form of FTD-4R tau (Spinelli et al., 2017).

Diagnosis and classification of PPA patients present a complex challenge that requires the integration of multimodal information, encompassing clinical, biological, and radiological features (Roytman et al., 2022). Concerning brain imaging alterations, several investigations have reported associations between language deficits and brain alterations in gray matter regions and white matter fiber bundles linking cortical areas associated with language (Agosta et al., 2015; Nigro et al., 2021; Tafuri et al., 2023). Moreover, svPPA showed a disruption of the ventral stream, impacting the occipito-temporal lobes (Galantucci et al., 2011; Agosta et al., 2013; Marcotte et al., 2017). Conversely, nfvPPA were characterized by damage to a more dorsal pathway, involving parieto-frontal regions (Galantucci et al., 2011; Agosta et al., 2013; Marcotte et al., 2017). Recently, morphometric and diffusivity features extracted in gray- and white-matter regions have also been used to develop diagnostic support systems to aid the clinical diagnosis and differentiation of patients with PPA (Agosta et al., 2015; Bisenius et al., 2017; Canu et al., 2019; Kim et al., 2019). While many studies have concentrated on creating automated systems using gray matter atrophy features (Agosta et al., 2015; Bisenius et al., 2017; Kim et al., 2019), only a handful of researchers have built classification models based on diffusion-based white matter damage (Agosta et al., 2015; Canu et al., 2019).

Within the field of diagnostic imaging, radiomics presents a novel approach of analysis, capable of unveiling imperceptible details within images (Gillies et al., 2016; Mayerhoefer et al., 2020). It quantifies alterations in texture within pathological regions of interest (ROIs). Consequently, numerous studies have employed the radiomics approach to uncover imaging biomarkers in cancers (Vial et al., 2018) and, more recently, to evaluate diagnosis and prognosis in other diseases, including neurodegenerative conditions (Salvatore et al., 2019; Feng and Ding, 2020; Tafuri et al., 2022a). In particular, classification models have been developed by extracting high-dimensional sets of radiomics measures in specific brain regions and then combining feature selectors and machine learning algorithms to distinguish between diagnostic categories (Feng et al., 2018; Ranjbar et al., 2019; Tafuri et al., 2022a,b; Rajagopalan et al., 2023). Despite the optimal performance obtained by these classification frameworks, however, the estimation of each feature contribution to the model’s classification is often unclear limiting the interpretability of the results. Thus, in recent years, the concept of explainability has received a lot of attention with the aim to understand the reasoning behind the model and in this way assess which information has the greatest impact on performance.

In the present study, we developed a radiomics-based classification approach to classify patients with PPA, conducting a secondary analysis from our previous work over the same population that evaluated the lateralized damage of structural white matter (Tafuri et al., 2023). In particular, 1st-order and 2nd-order statistic measures extracted from white matter regions and combined with clinical information were used as inputs to a tree-based algorithm to distinguish svPPA and nfvPPA from healthy controls, and to differentiate between PPA phenotypes. Moreover, the importance of features in the classification performance was evaluated by using a Shapley Additive Explanations (SHAP) method (Lundberg and Lee, 2017), a commonly employed approach widely applied in healthcare systems (Deshmukh and Merchant, 2020; Amoroso et al., 2023; Leandrou et al., 2023), and able to improve the interpretability of a machine learning model.

## 2 Materials and methods

### 2.1 Participants

Data were acquired from the Frontotemporal Lobar Degeneration Neuroimaging Initiative (FTLDNI) database (please visit http://memory.ucsf.edu/research). To minimize potential bias arising from different imaging protocols, we exclusively selected images acquired at the University of California, San Francisco, the largest recruiting center. In particular, out of the total sample from FTLDNI UCSF (37 nfvPPA; 34 svPPA, and 127 HC), we first considered subjects with a valid T1-weighted MRI sequence. Next, we randomly selected svPPA patients, nfvPPA patients, and healthy controls in order to have sex- and age-matched groups.

The primary goals of FTLDNI are to identify neuroimaging modalities and methods of analysis for tracking frontotemporal lobar degeneration and to assess the value of imaging vs. other markers in diagnostic roles. All patients underwent clinical, imaging, language, and neuropsychological examinations and met the current diagnostic criteria for Primary Progressive Aphasia (PPA) as defined by Gorno-Tempini et al. (2011). The Clinical Dementia Rating scale (CDR), with language subscore (CDR language), was administered to assess the global cognitive status (Morris, 1993; Knopman et al., 2008). Linguistic abilities were evaluated through the administration of tests such as the semantic verbal fluency (animal), the phonemic verbal fluency (d words) tests (Benton, 1969) and the total Boston Naming Test (BNT) (Kaplan et al., 1983). None of the controls had a history of neurologic or psychiatric illness (for more information, please refer to https://memory.ucsf.edu/research-trials/research/4rtni-2).

### 2.2 MRI data extraction

All subjects had a standard acquisition of MR images on a 3-T Siemens Trio Tim system equipped with a 12-channel head coil including whole-brain three-dimensional T1 MPRAGE (TR/TE = 2,300/2.9 ms, matrix = 240 × 256 × 160, isotropic voxels 1 mm^3^, slice thickness = 1 mm). An experienced neuroradiologist examined the images to exclude brain abnormalities, including lacunar and extensive cerebrovascular lesions.

We performed region segmentation from MRI using FreeSurfer 6.0 (Massachusetts General Hospital, Boston, MA) with the standard cross-sectional pipeline. The pre-processing steps conform scans to an isotropic voxel size of 1 mm^3^ followed by removal of non-brain tissue, bias correction, and segmentation into gray matter (GM), white matter (WM), and cerebrospinal fluid. Radiomics feature extraction was performed on the skull-stripped, non-uniform intensity-corrected image (*nu.mgz*). White-matter regions of interest (ROIs) were delineated using the FreeSurfer white matter parcellation approach (Salat et al., 2009), which classifies white matter based on the nearest cortical region of the Desikan-Killiany cortical atlas (Desikan et al., 2006). Consequently, we obtained 34 WM ROIs for each hemisphere to account for the asymmetric cerebral atrophy typically observed in PPA patients (Gorno-Tempini et al., 2011) (see Supplementary Table S2). Further details on these procedures have been documented in previous publications (Dale et al., 1999; Fischl et al., 2002, 2004).

**Figure 1:**
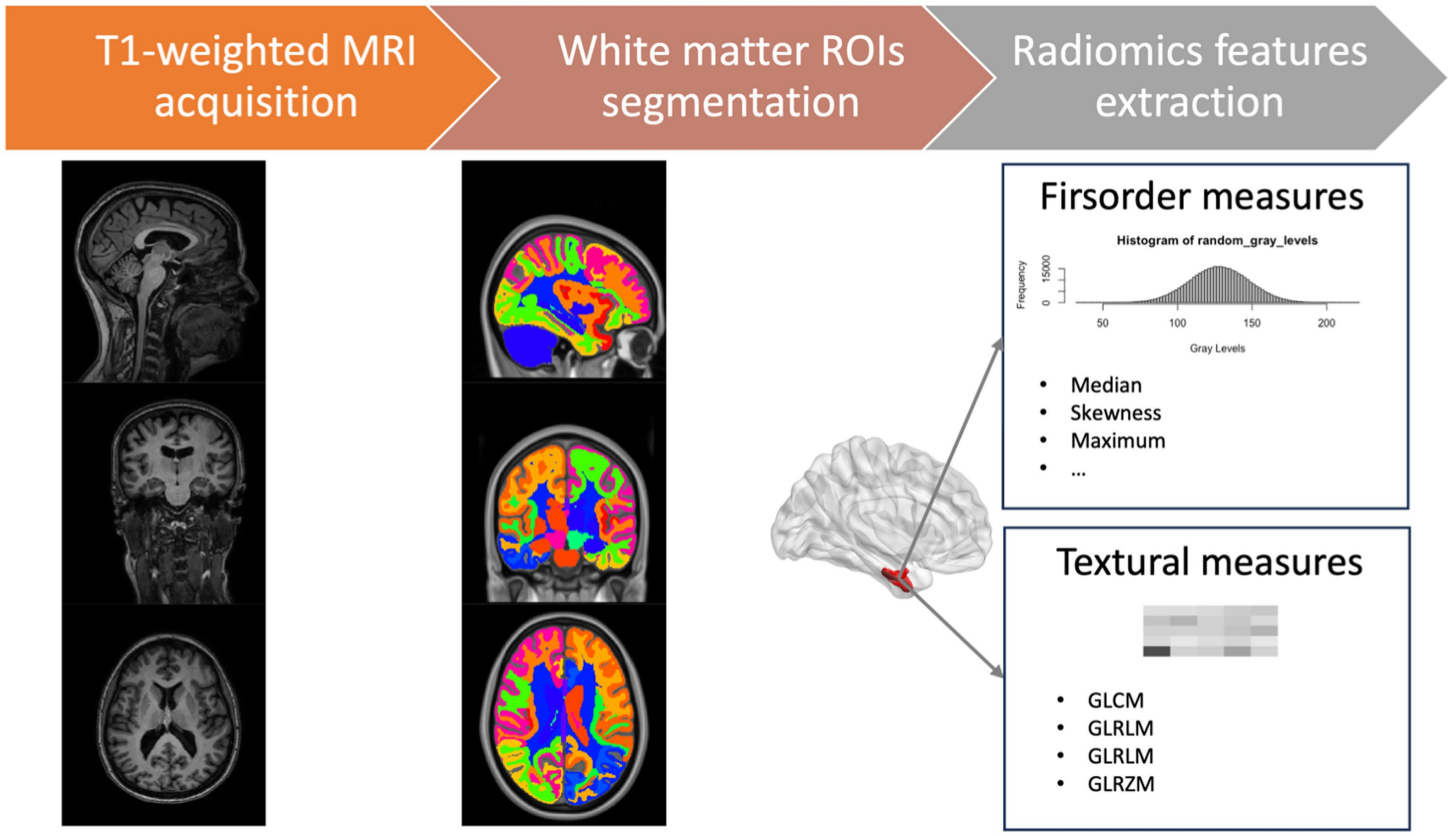
Schematic overview of the features extraction process. GLCM, gray level co-occurrence matrices; GLRLM, gray level run length matrices; GLDM, gray level dependence matrices; GLSZM, gray level size zone matrices.

For each ROI, we defined a set of 86 radiomic features in compliance with the Imaging Biomarker Standardization Initiative (IBSI) (Zwanenburg et al., 2020), comprising 16 first-order features to describe voxel intensity distribution within the image mask and 70 s-level textural measures to highlight the spatial distribution of voxels through five different matrices: 24 features from Gray Level Co-occurrence Matrices (GLCM), 16 from Gray Level Run Length Matrices (GLRLM), 14 measures from Gray Level Dependence Matrices (GLDM), and 16 features from Gray Level Size Zone Matrices (GLSZM) (detailed information about radiomics features is provided in Supplementary Table S1). In total, 5,848 radiomics measures were collected for each subject. A schematic overview of the features extraction process is reported in Figure 1. We used the Python package PyRadiomics 3.0 for extracting radiomics features (van Griethuysen et al., 2017).

### 2.3 Explainable machine learning pipeline

As a first step, we randomly split the data into training and test sets with a 70:30 proportion, ensuring that the samples were stratified to maintain the same label proportions in both the training and test folds. Then, we applied a feature selection method on the training set to prevent overfitting of the models. We conducted Pearson correlation analysis to eliminate redundancy between features, setting a cutoff coefficient of 0.9 (Schober et al., 2018; He et al., 2019; Bao et al., 2022; Leandrou et al., 2023). In particular, we identified the feature pair with the highest absolute correlation coefficient. Subsequently, we calculated the mean absolute correlation coefficient for each feature with all others, excluding the feature with the highest mean absolute correlation coefficient in each iteration. This iterative procedure continued until the pair-wise correlation coefficients among radiomic features dropped below 0.9 (Marzi et al., 2023). The remaining measures were then used for model development.

In this experiment, we chose the XGBoost classifier (Chen and Guestrin, 2016) as our baseline algorithm. XGBoost is the preferred choice among boosting techniques due to its outstanding classification performance, especially for imbalanced data. To be more specific, L1 and L2 regularization are responsible for managing sparsity and reducing overfitting. To optimize the model, we employed a randomized grid search technique with a stratified 5-fold cross-validation setting over the train dataset, running 60 iterations. The best model was determined by optimizing the learning rate (from 0.01 to 0.1), maximum depth (from 3 to 10), number of estimators (from 50 to 200), and subsampling (from 0.5 to 1) using the area under the receiver operating characteristic curve (AUC-ROC) as metric to evaluate the performances of the cross-validated model (see Supplementary Tables S4, S5 for the best hyperparameters). Finally, the importance of each feature was evaluated using Shapely Additive Explanations (SHAP) (Lundberg and Lee, 2017) based on Shapley values. This approach allows us to evaluate which measure has the most significant impact on the model’s performance. Specifically, it enables us to assess a feature’s impact on the entire training dataset, providing additional information beyond feature importance when considered in combination with other feature values, rather than as a single explainer.

### 2.4 Statistical analysis

Data for each group were explored using descriptive statistics as mean and standard deviation. We analyzed group differences in demographic and clinical data, using the chi-square test and Kruskal–Wallis analysis of variance, followed by post-hoc tests (Wilcoxon signed-rank test).

As regards classification analyses, we applied each trained model to the hold-out test set to evaluate its performance using various metrics, including sensitivity, specificity, balanced accuracy, precision, AUC-ROC, and F1 score. To evaluate and compare our radiomics-combined models with classical morphometric measures, we repeated the same analysis using volumetric data from each ROIs extracted by FreeSurfer toolbox.

## 3 Results

The final cohort of the study included 109 subjects: 31 svPPA, 25 nfvPPA, and 53 healthy controls (HC), with sex and age matching. Concerning clinical data, PPA groups differed significantly from HC subjects (see Table 1). Furthermore, svPPA reported a significant impairment of performances in Boston Naming Test respect to nfvPPA patients (*p*-values <0.001) while they performed better than the latter in the phonemic verbal fluency (d words) test (*p*-value <0.001).

**Table 1:** Demographic and clinical/cognitive information.

	HC *n* = 53	nfvPPA *n* = 25	svPPA *n* = 31	Kruskal–Wallis/*χ*^2^ test	Post-hoc
	Mean or # (SD or %)	Mean or # (SD or %)	Mean or # (SD or %)	*p*	
Age, years	64.11 (6.33)	66.56 (6.66)	62.97 (6.43)	0.113	–
Female	30 (56.6)	14 (56.0)	15 (48.4)	0.749	–
Education, years	21.96 (19.13)	15.80 (2.60)	19.06 (15.08)	0.263	–
CDR	0.03 (0.12)*	0.44 (0.34)	0.65 (0.32)	<0.001	HCvsnfvPPA, svPPA, <0.001
CDR language	0.00 (0.00)*	1.25 (0.77)	0.95 (0.51)	<0.001	HCvsnfvPPA, svPPA, <0.001
Verbal Fluency-Animal	23.53 (5.14)	11.88 (8.69)	9.03 (4.15)	<0.001	HCvsnfvPPA, svPPA, <0.001
Verbal Fluency-d words	15.87 (4.13)	6.60 (6.18)	8.97 (4.48)	<0.001	HCvsnfvPPA, svPPA, <0.001 nfvPPA vs. svPPA, 0.01
Boston Naming Test	14.32 (0.89)	12.52 (2.45)	5.71 (3.53)	<0.001	HCvsnfvPPA, svPPA, <0.001 nfvPPA vs. svPPA, <0.001

HC, healthy controls; nfvPPA, non-fluent variant of primary progressive aphasia; svPPA, semantic variant of primary progressive aphasia; CDR, clinical dementia rating scale.

*Data available for 24 out of 53 healthy controls.

All XGBoost binary models were trained over 70% of the samples (37 HC, 21 svPPA, and 17 nfvPPA) and evaluated over the remaining unseen 30% of the dataset (including 16 HC, 10 svPPA, and 8 nfvPPA).

As first step, we checked the performances of the models trained only considering clinical/cognitive variables. The results are reported in Table 2 showing that the performances of the comparisons between PPA patients and HC achieved optimal values (svPPA versus HC had balanced accuracy of 0.95, sensitivity of 1 and specificity of 0.9, while nfvPPA versus HC had balanced accuracy of 0.937, sensitivity of 1 and specificity of 0.875). By contrast, the discrimination between semantic and non-fluent/agrammatic variants of PPA remained suboptimal (balanced accuracy of 0.771, sensitivity of 0.667, and specificity of 0.875).

**Table 2:** XGBoost classification performance of clinical/cognitive model between groups on test set.

Metrics	HC vs. svPPA	HC vs. nfvPPA	svPPA vs. nfvPPA
Sensitivity	1	1	0.667
Specificity	0.9	0.875	0.875
Balanced Accuracy	0.95	0.937	0.771
F1-score	0.947	0.933	0.778
AUC-ROC	0.987	0.984	0.792
Precision	1	1	0.7

HC, healthy controls; nfvPPA, non-fluent variant of primary progressive aphasia; svPPA, semantic variant of primary progressive aphasia; AUC-ROC, area under the curve of the receiver operating characteristic.

Concerning radiomics analysis, the selected radiomics features at each training step, combined with clinical/cognitive information, were then used as input for classification analyses. As reported in Table 3, the XGBoost model confirmed optimal results in distinguishing svPPA and nfvPPA patients from HC (svPPA versus HC had balanced accuracy of 0.95, sensitivity of 1 and specificity of 0.9, while nfvPPA versus HC had balanced accuracy of 0.937, sensitivity of 1 and specificity of 0.875). Furthermore, the discriminations between svPPA and nfvPPA patients achieved balanced accuracy of 0.937, sensitivity of 1 and specificity of 0.875. Of note, in Supplementary Table S3 we reported the performances of volumetric-combined model. Even if classical morphometric features reached optimal results, radiomics model optimized all the performances of classification.

**Table 3:** XGBoost classification performance of clinical/cognitive + radiomics model between groups on test set.

Metrics	HC vs. svPPA	HC vs. nfvPPA	svPPA vs. nfvPPA
Sensitivity	1	1	1
Specificity	0.9	0.875	0.875
Balanced accuracy	0.95	0.937	0.937
F1-score	0.947	0.933	0.933
AUC-ROC	0.987	0.984	1
Precision	1	1	1

HC, healthy controls; nfvPPA, non-fluent variant of primary progressive aphasia; svPPA, semantic variant of primary progressive aphasia; AUC-ROC, area under the curve of the receiver operating characteristic.

**Figure 2:**
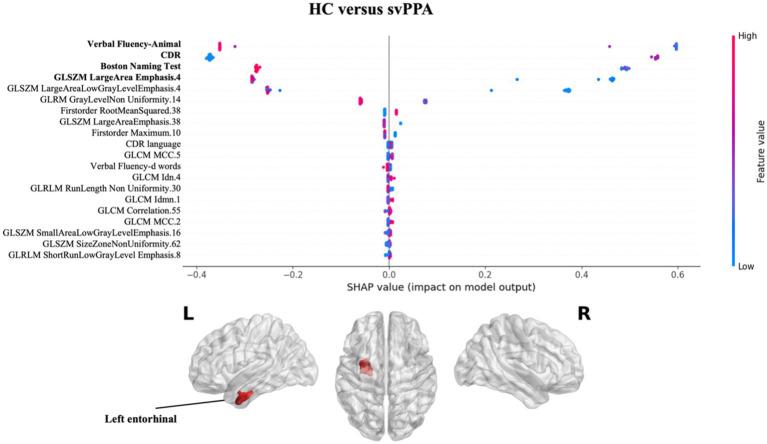
Impact of radiomics features on the classification of HC vs. svPPA group. Beeswarm plot reporting radiomic measures explainability for svPPA classification model. Each point represents the SHAP value per subject. Red and blue report higher to lower values of the feature. HC, healthy controls; svPPA, semantic variant of primary progressive aphasia; GLCM, gray level co-occurrence matrices; GLRLM, gray level run length matrices; GLDM, gray level dependence matrices; GLSZM, gray level size zone matrices.

Regarding the contribution of each measures (clinical/cognitive and radiomics) in classification performance, the explainability analysis for svPPA classification (see Figure 2 and Supplementary Table S2) revealed that language deficits (verbal fluency-animal and BNT tests) together with a compromised clinical condition (CDR) of patients respect to healthy subjects had the greater impact in classification. Nonetheless, radiomics measures from the white matter region near the left entorhinal cortex had a significant impact on predicting svPPA syndrome compared to control subjects, also corresponding to lower values of radiomic features for patients.

**Figure 3:**
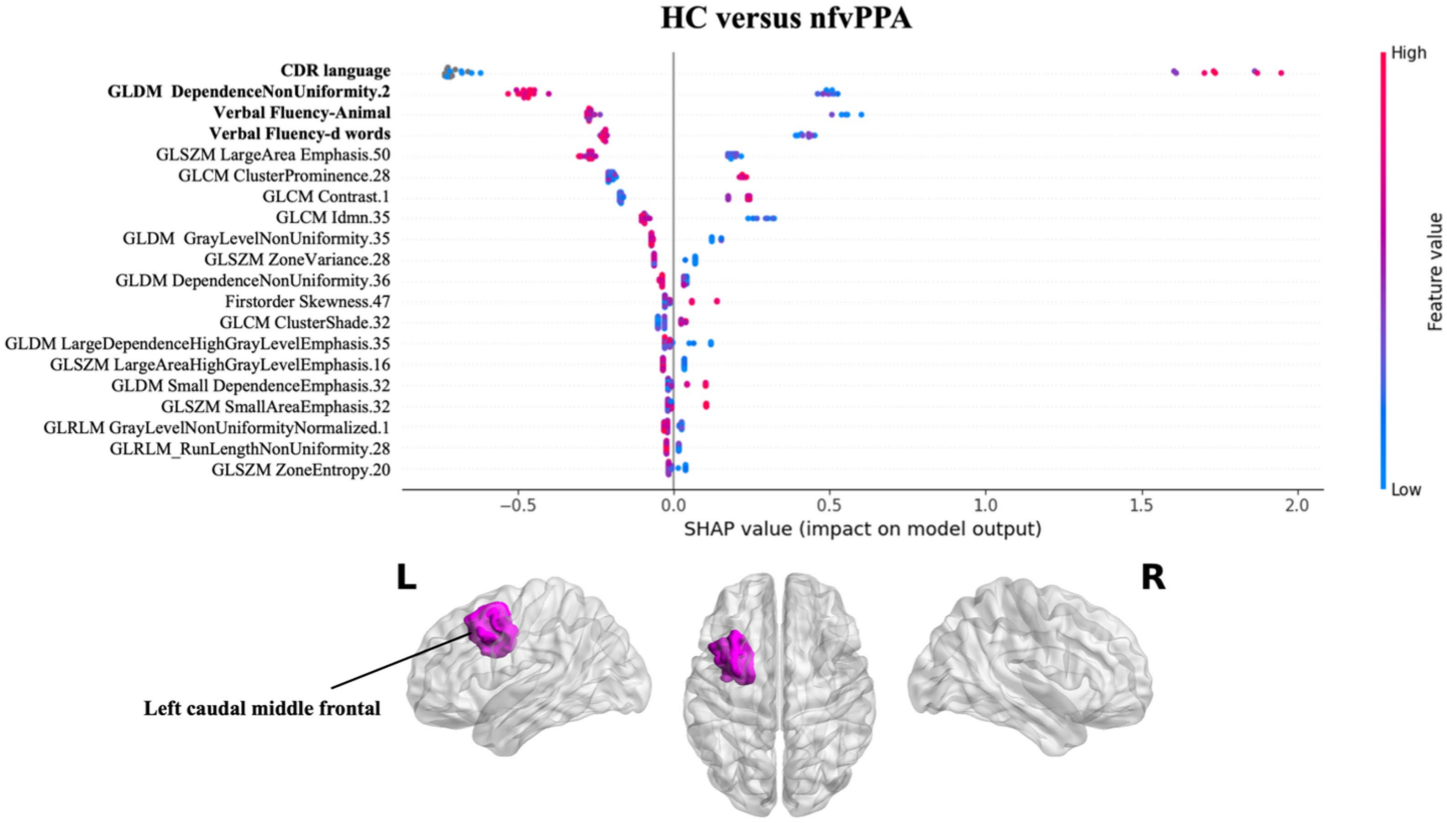
Impact of radiomics features on the classification of HC vs. nfvPPA group. Beeswarm plot reporting radiomic measures explainability for nfvPPA classification model. Each point represents the SHAP value per subject. Red and blue report higher to lower values of the feature. HC, healthy controls; nfvPPA, non-fluent variant of primary progressive aphasia; GLCM, gray level co-occurrence matrices; GLRLM, gray level run length matrices; GLDM, gray level dependence matrices; GLSZM, gray level size zone matrices.

Figure 3 presents the results of SHAP values for classifying nfvPPA patients compared to HC. Similarly to svPPA classification, the greatest impact on the classification was observed for the linguistic scores (CDR language and verbal fluency test), together with the radiomics feature of the left white matter near the caudal middle frontal gyrus, with lower values for patients compared to controls.

**Figure 4:**
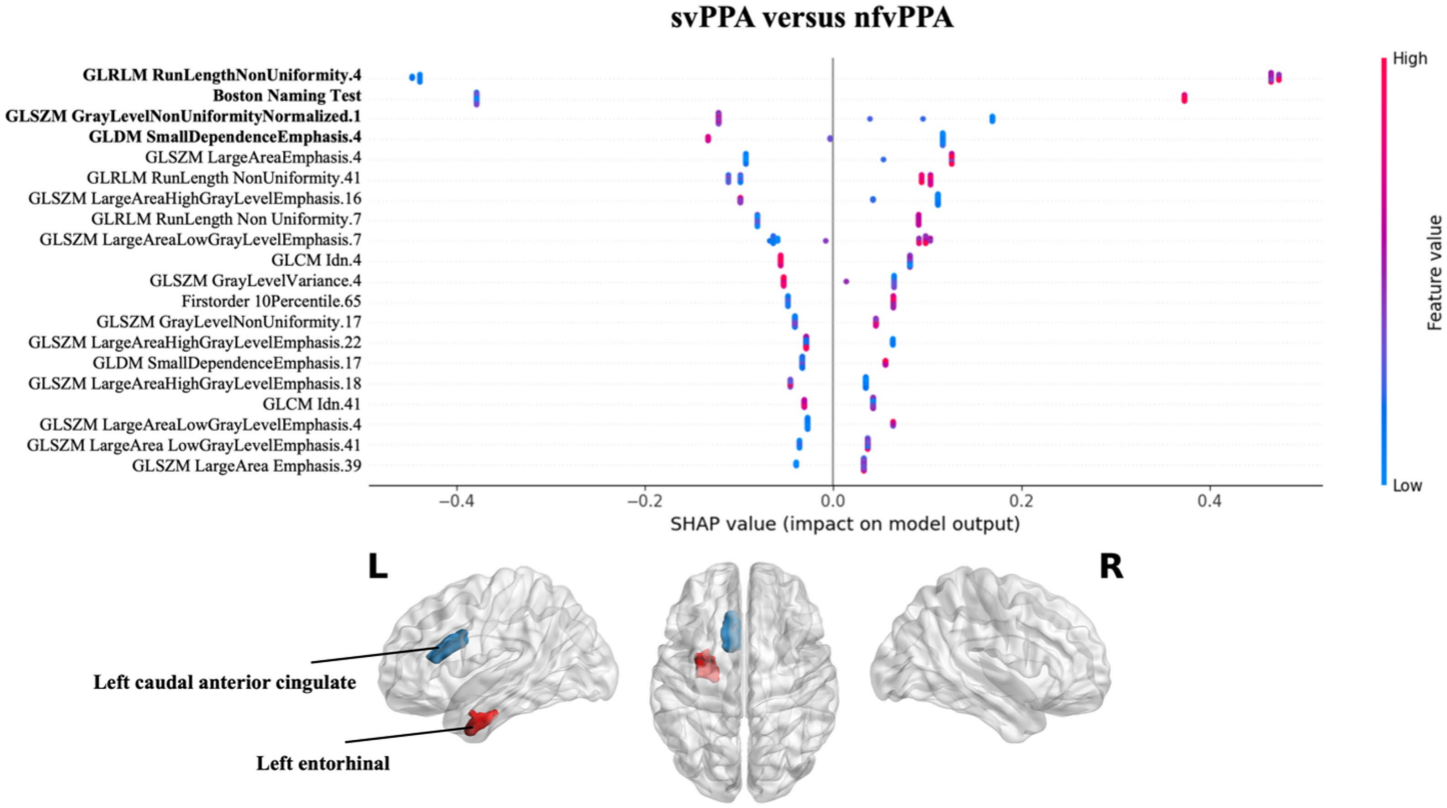
Impact of radiomics features on the classification of svPPA vs. nfvPPA group. Beeswarm plot reporting radiomic measures explainability for svPPA versus nfvPPA classification model. Each point represents the SHAP value per subject. Red and blue report higher to lower values of the feature. HC, healthy controls; svPPA, semantic variant of primary progressive aphasia; nfvPPA, non-fluent variant of primary progressive aphasia; GLCM, gray level co-occurrence matrices; GLRLM, gray level run length matrices; GLDM, gray level dependence matrices; GLSZM, gray level size zone matrices.

Finally, the nfvPPA classification respect to svPPA highlighted that the radiomics features of the white matter of the left entorhinal together with the Boston Naming Test score had greatest predictive power for the model. In particular higher values of GLRLM RunLenghtNonUniformity of the left entorhinal were high predictive of nfvPPA syndrome (see Figure 4).

## 4 Discussion

In the present study, clinical information and radiomic features in white matter regions were used for discriminating PPA patients. Radiomics features substantially improved the performance of classification between patient groups with respect to solely clinical/cognitive scores. Concerning the features explainability, the SHAP method highlighted the greater impact of the left entorhinal cortex in distinguishing between svPPA and nfvPPA patients. On the contrary, the contribution of radiomics in classifying patients from controls was limited. Indeed, the SHAP method showed the stronger impact of clinical/cognitive scores in discriminate PPA patients respect to controls.

The performance of our models are in line with previous studies using MRI data to support the clinical diagnosis of patients with PPA (Agosta et al., 2015; Bisenius et al., 2017; Lampe et al., 2022; Tafuri et al., 2022a). Specifically, when classifying svPPA against HC, our model achieved an accuracy of 95%, which is comparable to the results obtained using gray matter radiomic measures (Tafuri et al., 2022a), or features such as cortical thickness and Diffusion Tensor Imaging (DTI) [as observed in studies by Bisenius et al., 2017 and Agosta et al., 2015]. Furthermore, the analysis of explainability through SHAP values confirmed that the left temporal lobe, and in particular the entorhinal cortex, was the most affected region in svPPA (Chan et al., 2001; Agosta et al., 2015; Bisenius et al., 2017; Lampe et al., 2022), not only in terms of gray matter but also in white matter.

As observed in previous studies, the classification of nfvPPA is a more challenging and difficult task using imaging data. Specifically, a recent work by Lampe et al. (2022) reported low performances for classification of nfvPPA patients for a multi-syndrome model based on a multi-centric MRI dataset. Our radiomics-integrated model demonstrated an optimal accuracy of 93.7% when discriminating these patients from HC. However, when considering the impact of measures on the model, we found that the most impactful variables corresponded to clinical scores, confirming the low contribution of imaging markers in distinguishing nfvPPA patients from healthy controls.

Regarding the differentiation between the two PPA variants, our combined model achieved a diagnostic accuracy of 93.7% on the test set. This result overcomes the state-of-the-art performances achieved using only classical morphometry measurements (Agosta et al., 2015; Bisenius et al., 2017; Kim et al., 2019; Lampe et al., 2022) and radiomics on gray matter ROIs (Tafuri et al., 2022a), in conjunction with machine learning systems like Support Vector Machine, Random Forest, or Linear Discriminant Analysis. It’s worth noting that, contrary to the comparison between pathological and healthy subjects, clinical/cognitive variables were unable to correctly identify the PPA phenotype achieving a suboptimal accuracy of classification of the 77.1%. On the contrary, the combination with radiomics measures reached the best performance of 93.7% of accuracy and the most influential feature in the model was associated with the left white matter of the entorhinal cortex. This region is a distinctive characteristic of the svPPA variant, as previously indicated in radiomics findings (Tafuri et al., 2022a), and is morphologically linked to more pronounced cortical thinning compared to nfvPPA (Agosta et al., 2015).

The current study has some limitations that need to be addressed. Firstly, the study is based on a modest sample size in the context of a machine learning approach, which we addressed by implementing a cross-validation setting. Consequently, future approaches should prioritize an expanded and more representative data sample encompassing the entire spectrum of PPA, also in combination with more reliable features selection methods to guarantee maximal generalizability. Secondly, radiomics features in white matter regions were extracted from T1-weighted MR images, excluding the possibility of conducting a comparative analysis using diffusivity information. Therefore, further studies should be conducted to explore the usefulness of DTI radiomics in differentiating PPA patients. Another potential limitation is the absence of the biological confirmation, such as cerebrospinal fluid (CSF) or amyloid PET samples. In the future, it would be beneficial to incorporate biological assessment and evaluate various classification algorithms using a multicenter dataset. Thirdly, we conducted Pearson correlation analyses to eliminate feature redundancy before model training. Although this approach is typically employed to address the inherent multicollinearity of radiomics measures, it is possible that similar classification metric values may be obtained by considering the excluded radiomics features. Finally, longitudinal studies are required to assess whether WM radiomics features could also be used to develop predictive models of clinical-pathological progression.

## 5 Conclusion

Our study provides new evidence for the usefulness of radiomics features in classifying patients with neurodegenerative diseases. In particular, the results of this study show that texture properties of the brain’s white matter, based on conventional T1-weighted MR images, substantially improve the classification performances opening the way to new potential imaging biomarkers to classify PPA patients. In particular, radiomics features extracted in the white matter near the left entorhinal cortex may help to the differentiate nfvPPA from svPPA patients.

## Data availability statement

The raw data supporting the conclusions of this article will be made available by the authors, without undue reservation.

## Ethics statement

Ethical approval was not required for the studies involving humans in accordance with the local legislation and institutional requirements. The studies were conducted in accordance with the local legislation and institutional requirements. The participants provided their written informed consent to participate in this study.

## Frontotemporal Lobar Degeneration Neuroimaging Initiative

Data used in the preparation of this article were obtained from the Frontotemporal Lobar Degeneration Neuroimaging Initiative (FTLDNI) database (http://4rtni-ftldni.ini.usc.edu/). The investigators at NIFD/FTLDNI contributed to the design and implementation of FTLDNI and/or provided data but did not participate in the analysis or writing of this report (unless otherwise listed).

## Author contributions

BT: Conceptualization, Formal analysis, Methodology, Software, Visualization, Writing – original draft, Writing – review & editing. RB: Data curation, Resources, Supervision, Writing – review & editing. SN: Conceptualization, Methodology, Project administration, Supervision, Validation, Writing – review & editing. GL: Conceptualization, Funding acquisition, Investigation, Project administration, Resources, Supervision, Writing – review & editing.

## References

[ref1] Agosta F. Ferraro P. M. Canu E. Copetti M. Galantucci S. Magnani G.. (2015). Differentiation between subtypes of primary progressive aphasia by using cortical thickness and diffusion-tensor MR imaging measures. Radiology 276, 219–227. doi: 10.1148/radiol.15141869, PMID: 25734554

[ref2] Agosta F. Galantucci S. Canu E. Cappa S. F. Magnani G. Franceschi M.. (2013). Disruption of structural connectivity along the dorsal and ventral language pathways in patients with nonfluent and semantic variant primary progressive aphasia: a DT MRI study and a literature review. Brain Lang. 127, 157–166. doi: 10.1016/j.bandl.2013.06.003, PMID: 23890877

[ref3] Amoroso N. Quarto S. La Rocca M. Tangaro S. Monaco A. Bellotti R. (2023). An eXplainability artificial intelligence approach to brain connectivity in Alzheimer’s disease. Front. Aging Neurosci. 15:1238065. doi: 10.3389/fnagi.2023.1238065, PMID: 37719873 PMC10501457

[ref4] Bao D. Liu Z. Geng Y. Li L. Xu H. Zhang Y.. (2022). Baseline MRI-based radiomics model assisted predicting disease progression in nasopharyngeal carcinoma patients with complete response after treatment. Cancer Imaging 22:10. doi: 10.1186/s40644-022-00448-4, PMID: 35090572 PMC8800208

[ref5] Benton A. L. (1969). Development of a multilingual aphasia battery progress and problems. J Neurol Sci 9, 39–48. doi: 10.1016/0022-510x(69)90057-4, PMID: 5820858

[ref6] Bisenius S. Mueller K. Diehl-Schmid J. Fassbender K. Grimmer T. Jessen F.. (2017). Predicting primary progressive aphasias with support vector machine approaches in structural MRI data. Neuroimage Clin 14, 334–343. doi: 10.1016/j.nicl.2017.02.003, PMID: 28229040 PMC5310935

[ref7] Canu E. Agosta F. Imperiale F. Fontana A. Caso F. Spinelli E. G.. (2019). Added value of multimodal MRI to the clinical diagnosis of primary progressive aphasia variants. Cortex 113, 58–66. doi: 10.1016/j.cortex.2018.11.025, PMID: 30605869

[ref8] Chan D. Fox N. C. Scahill R. I. Crum W. R. Whitwell J. L. Leschziner G.. (2001). Patterns of temporal lobe atrophy in semantic dementia and Alzheimer’s disease. Ann. Neurol. 49, 433–442. doi: 10.1002/ana.92 11310620

[ref9] Chen T. Guestrin C. (2016). XGBoost: a scalable tree boosting system. In Proceedings of the 22nd ACM SIGKDD international conference on knowledge discovery and data mining KDD ‘16. New York, NY, USA: Association for Computing Machinery, 785–794

[ref10] Coyle-Gilchrist I. T. S. Dick K. M. Patterson K. Vázquez Rodríquez P. Wehmann E. Wilcox A.. (2016). Prevalence, characteristics, and survival of frontotemporal lobar degeneration syndromes. Neurology 86, 1736–1743. doi: 10.1212/WNL.0000000000002638, PMID: 27037234 PMC4854589

[ref11] Dale A. M. Fischl B. Sereno M. I. (1999). Cortical surface-based analysis. I. Segmentation and surface reconstruction. NeuroImage 9, 179–194. doi: 10.1006/nimg.1998.0395 9931268

[ref12] Deshmukh F. Merchant S. S. (2020). Explainable machine learning model for predicting GI bleed mortality in the intensive care unit. Am. J. Gastroenterol. 115, 1657–1668. doi: 10.14309/ajg.0000000000000632, PMID: 32341266

[ref13] Desikan R. S. Ségonne F. Fischl B. Quinn B. T. Dickerson B. C. Blacker D.. (2006). An automated labeling system for subdividing the human cerebral cortex on MRI scans into gyral based regions of interest. NeuroImage 31, 968–980. doi: 10.1016/j.neuroimage.2006.01.021, PMID: 16530430

[ref14] Feng Q. Chen Y. Liao Z. Jiang H. Mao D. Wang M.. (2018). Corpus callosum radiomics-based classification model in Alzheimer’s disease: a case-control study. Front. Neurol. 9:618. doi: 10.3389/fneur.2018.00618, PMID: 30093881 PMC6070743

[ref15] Feng Q. Ding Z. (2020). MRI Radiomics classification and prediction in Alzheimer’s disease and mild cognitive impairment: a review. Curr. Alzheimer Res. 17, 297–309. doi: 10.2174/1567205017666200303105016, PMID: 32124697

[ref16] Fischl B. Salat D. H. Busa E. Albert M. Dieterich M. Haselgrove C.. (2002). Whole brain segmentation: automated labeling of neuroanatomical structures in the human brain. Neuron 33, 341–355. doi: 10.1016/s0896-6273(02)00569-x 11832223

[ref17] Fischl B. van der Kouwe A. Destrieux C. Halgren E. Ségonne F. Salat D. H.. (2004). Automatically parcellating the human cerebral cortex. Cereb. Cortex 14, 11–22. doi: 10.1093/cercor/bhg087 14654453

[ref18] Galantucci S. Tartaglia M. C. Wilson S. M. Henry M. L. Filippi M. Agosta F.. (2011). White matter damage in primary progressive aphasias: a diffusion tensor tractography study. Brain 134, 3011–3029. doi: 10.1093/brain/awr099, PMID: 21666264 PMC3187537

[ref19] Gillies R. J. Kinahan P. E. Hricak H. (2016). Radiomics: images are more than pictures, they are data. Radiology 278, 563–577. doi: 10.1148/radiol.2015151169, PMID: 26579733 PMC4734157

[ref20] Gorno-Tempini M. L. Hillis A. E. Weintraub S. Kertesz A. Mendez M. Cappa S. F.. (2011). Classification of primary progressive aphasia and its variants. Neurology 76, 1006–1014. doi: 10.1212/WNL.0b013e31821103e6, PMID: 21325651 PMC3059138

[ref21] He L. Huang Y. Yan L. Zheng J. Liang C. Liu Z. (2019). Radiomics-based predictive risk score: a scoring system for preoperatively predicting risk of lymph node metastasis in patients with resectable non-small cell lung cancer. Chin. J. Cancer Res. 31, 641–652. doi: 10.21147/j.issn.1000-9604.2019.04.08, PMID: 31564807 PMC6736655

[ref22] Kaplan E. Goodglass H. Weintraub S. (1983). Boston naming test Lea & Febiger.

[ref23] Kim J. P. Kim J. Park Y. H. Park S. B. Lee J. S. Yoo S.. (2019). Machine learning based hierarchical classification of frontotemporal dementia and Alzheimer’s disease. NeuroImage: Clinical 23:101811. doi: 10.1016/j.nicl.2019.101811 30981204 PMC6458431

[ref24] Knopman D. S. Kramer J. H. Boeve B. F. Caselli R. J. Graff-Radford N. R. Mendez M. F.. (2008). Development of methodology for conducting clinical trials in frontotemporal lobar degeneration. Brain 131, 2957–2968. doi: 10.1093/brain/awn234, PMID: 18829698 PMC2725027

[ref25] Lampe L. Niehaus S. Huppertz H.-J. Merola A. Reinelt J. Mueller K.. (2022). Comparative analysis of machine learning algorithms for multi-syndrome classification of neurodegenerative syndromes. Alzheimers Res. Ther. 14:62. doi: 10.1186/s13195-022-00983-z, PMID: 35505442 PMC9066923

[ref26] Leandrou S. Lamnisos D. Bougias H. Stogiannos N. Georgiadou E. Achilleos K. G.. (2023). A cross-sectional study of explainable machine learning in Alzheimer’s disease: diagnostic classification using MR radiomic features. Front. Aging Neurosci. 15:e1149871. doi: 10.3389/fnagi.2023.1149871, PMID: 37358951 PMC10285704

[ref27] Leyton C. E. Villemagne V. L. Savage S. Pike K. E. Ballard K. J. Piguet O.. (2011). Subtypes of progressive aphasia: application of the international consensus criteria and validation using β-amyloid imaging. Brain 134, 3030–3043. doi: 10.1093/brain/awr216, PMID: 21908392

[ref28] Lundberg S. M. Lee S.-I. (2017). A unified approach to interpreting model predictions. In advances in neural information processing systems (Curran associates, Inc.). Available at: https://papers.nips.cc/paper_files/paper/2017/hash/8a20a8621978632d76c43dfd28b67767-Abstract.html (Accessed September 21, 2023).

[ref29] Marcotte K. Graham N. L. Fraser K. C. Meltzer J. A. Tang-Wai D. F. Chow T. W.. (2017). White matter disruption and connected speech in non-fluent and semantic variants of primary progressive aphasia. Dement Geriatr Cogn Dis Extra 7, 52–73. doi: 10.1159/000456710, PMID: 28611820 PMC5465709

[ref30] Marzi C. Marfisi D. Barucci A. Del Meglio J. Lilli A. Vignali C.. (2023). Collinearity and dimensionality reduction in Radiomics: effect of preprocessing parameters in hypertrophic cardiomyopathy magnetic resonance T1 and T2 mapping. Bioengineering 10:80. doi: 10.3390/bioengineering10010080, PMID: 36671652 PMC9854492

[ref31] Mayerhoefer M. E. Materka A. Langs G. Häggström I. Szczypiński P. Gibbs P.. (2020). Introduction to Radiomics. J. Nucl. Med. 61, 488–495. doi: 10.2967/jnumed.118.222893, PMID: 32060219 PMC9374044

[ref32] Mesulam M. M. (1982). Slowly progressive aphasia without generalized dementia. Ann. Neurol. 11, 592–598. doi: 10.1002/ana.410110607 7114808

[ref33] Morris J. C. (1993). The clinical dementia rating (CDR): current version and scoring rules. Neurology 43, 2412–2414. doi: 10.1212/wnl.43.11.2412-a 8232972

[ref34] Nigro S. Tafuri B. Urso D. De Blasi R. Cedola A. Gigli G.. (2021). Altered structural brain networks in linguistic variants of frontotemporal dementia. Brain Imaging Behav. 16:1113. doi: 10.1007/s11682-021-00560-2, PMID: 34755293 PMC9107413

[ref35] Rajagopalan V. Chaitanya K. G. Pioro E. P. (2023). Quantitative brain MRI metrics distinguish four different ALS phenotypes: a machine learning based study. Diagnostics 13:1521. doi: 10.3390/diagnostics13091521, PMID: 37174914 PMC10177762

[ref36] Ranjbar S. Velgos S. N. Dueck A. C. Geda Y. E. Mitchell J. R. Alzheimer’s Disease Neuroimaging Initiative (2019). Brain MR Radiomics to differentiate cognitive disorders. J. Neuropsychiatry Clin. Neurosci. 31, 210–219. doi: 10.1176/appi.neuropsych.17120366, PMID: 30636564 PMC6626704

[ref37] Roytman M. Chiang G. C. Gordon M. L. Franceschi A. M. (2022). Multimodality imaging in primary progressive aphasia. AJNR Am. J. Neuroradiol. 43, 1230–1243. doi: 10.3174/ajnr.A7613, PMID: 36007947 PMC9451618

[ref38] Salat D. Lee S. van der Kouwe A. Greve D. Fischl B. Rosas H. (2009). Age-associated alterations in cortical gray and white matter signal intensity and gray to white matter contrast. NeuroImage 48, 21–28. doi: 10.1016/j.neuroimage.2009.06.074, PMID: 19580876 PMC2750073

[ref39] Salvatore C. Castiglioni I. Cerasa A. (2019). Radiomics approach in the neurodegenerative brain. Aging Clin. Exp. Res. 33, 1709–1711. doi: 10.1007/s40520-019-01299-z, PMID: 31428998

[ref40] Schober P. Boer C. Schwarte L. A. (2018). Correlation coefficients: appropriate use and interpretation. Anesth. Analg. 126, 1763–1768. doi: 10.1213/ANE.0000000000002864 29481436

[ref41] Spinelli E. G. Mandelli M. L. Miller Z. A. Santos-Santos M. A. Wilson S. M. Agosta F.. (2017). Typical and atypical pathology in primary progressive aphasia variants. Ann. Neurol. 81, 430–443. doi: 10.1002/ana.24885, PMID: 28133816 PMC5421819

[ref42] Tafuri B. Filardi M. Urso D. De Blasi R. Rizzo G. Nigro S.. (2022a). Radiomics model for frontotemporal dementia diagnosis using T1-weighted MRI. Front. Neurosci. 16:828029. doi: 10.3389/fnins.2022.828029, PMID: 35794955 PMC9251132

[ref43] Tafuri B. Filardi M. Urso D. Gnoni V. De Blasi R. Nigro S.. (2023). Asymmetry of radiomics features in the white matter of patients with primary progressive aphasia. Front. Aging Neurosci. 15:1120935. doi: 10.3389/fnagi.2023.1120935, PMID: 37213534 PMC10196268

[ref44] Tafuri B. Lombardi A. Nigro S. Urso D. Monaco A. Pantaleo E.. (2022b). The impact of harmonization on radiomic features in Parkinson’s disease and healthy controls: a multicenter study. Front. Neurosci. 16:1012287. doi: 10.3389/fnins.2022.1012287, PMID: 36300169 PMC9589497

[ref45] Tee B. L. Gorno-Tempini M. L. (2019). Primary progressive aphasia: a model for neurodegenerative disease. Curr. Opin. Neurol. 32, 255–265. doi: 10.1097/WCO.0000000000000673, PMID: 30694922 PMC6602793

[ref46] van Griethuysen J. J. M. Fedorov A. Parmar C. Hosny A. Aucoin N. Narayan V.. (2017). Computational radiomics system to decode the radiographic phenotype. Cancer Res. 77, e104–e107. doi: 10.1158/0008-5472.CAN-17-0339 29092951 PMC5672828

[ref47] Vial A. Stirling D. Field M. Ros M. Ritz C. Carolan M.. (2018). The role of deep learning and radiomic feature extraction in cancer-specific predictive modelling: a review. Transl. Cancer Res. 7, 803–816. doi: 10.21037/tcr.2018.05.02

[ref48] Zwanenburg A. Leger S. Vallières M. Löck S. (2020). Image biomarker standardisation initiative. Radiology 295, 328–338. doi: 10.1148/radiol.2020191145, PMID: 32154773 PMC7193906

