# Supplementary material for: Explainable machine learning radiomics model for Primary Progressive Aphasia classification
Source: Front Syst Neurosci. 2024 Mar 18;18:1324437. doi: 10.3389/fnsys.2024.1324437 (PMC10982515; doi:10.3389/fnsys.2024.1324437)
Supplement: Supplementary file 1 [file Table_1.DOCX]

**Supplementary materials**

Table S1. Detailed features extracted from each region-of-interest.

| Type | Measure |
| --- | --- |
| First order | *Interquartile Range* |
|  | *Skewness* |
|  | *Uniformity* |
|  | *Median* |
|  | *Robust Mean Absolute Deviation* |
|  | *Maximum* |
|  | *Root Mean Squared* |
|  | *90° Percentile* |
|  | *Minimum* |
|  | *Entropy* |
|  | *Range* |
|  | *Variance* |
|  | *10° Percentile* |
|  | *Kurtosis* |
|  | *Mean* |
|  | *Mean Absolute Deviation* |
| GLDM | *Gray Level Variance* |
| (Gray Level Dependence matrix) | *High Gray Level Emphasis* |
|  | *Dependence Entropy* |
|  | *Dependence Non Uniformity* |
|  | *Gray Level Non Uniformity* |
|  | *Small Dependence Emphasis* |
|  | *Small Dependence High Gray Level Emphasis* |
|  | *Dependence Non Uniformity Normalized* |
|  | *Large Dependence Emphasis* |
|  | *Large Dependence Low Gray Level Emphasis* |
|  | *Dependence Variance* |
|  | *Large Dependence High Gray Level Emphasis* |
|  | *Small Dependence Low Gray Level Emphasis* |
|  | *Low Gray Level Emphasis* |
| GLCM | *Joint Average* |
| (Gray level Cooccurence matrix) | *Sum Average* |
|  | *Joint Entropy* |
|  | *Cluster Shade* |
|  | *Maximum Probability* |
|  | *Inverse Difference Moment Normalized* |
|  | *Joint Energy* |
|  | *Contrast* |
|  | *Difference Entropy* |
|  | *Inverse Variance* |
|  | *Difference Variance* |
|  | *Inverse Difference Normalized* |
|  | *Inverse Difference Moment* |
|  | *Correlation* |
|  | *Autocorrelation* |
|  | *Sum Entropy* |
|  | *Maximal Correlation Coefficient* |
|  | *Sum Squared* |
|  | *Cluster Prominence* |
|  | *Informational Measure of Correlation 2* |
|  | *Informational Measure of Correlation 1* |
|  | *Difference Average* |
|  | *Inverse Difference* |
|  | *Cluster Tendency* |
| GLRLM | *Short Run Low Gray Level Emphasis* |
| (gray level run length matrix) | *Gray Level Variance* |
|  | *Low Gray Level Non Uniformity Normalized* |
|  | *Gray Level Non Uniformity Normalized* |
|  | *Run Variance* |
|  | *Gray Level Non Uniformity* |
|  | *Long Run Emphasis* |
|  | *Short Run High Gray Level Emphasis* |
|  | *Run Length Non Uniformity* |
|  | *Short Run Non Uniformity* |
|  | *Long Run High Gray Level Emphasis* |
|  | *Run Percentage* |
|  | *Long Run Low Gray Level Emphasis* |
|  | *Run Entropy* |
|  | *High Gray Level Run Emphasis* |
|  | *Run Length Non Uniformity Normalized* |
| GLSZM | *Gray Level Variance* |
| (gray level size zone matrix) | *Zone Variance* |
|  | *Gray Level Non Uniformity Normalized* |
|  | *Size Zone Non Uniformity Normalized* |
|  | *Size Zone Non Uniformity* |
|  | *Gray Level Non Uniformity* |
|  | *Large Area Emphasis* |
|  | *Small Area High Gray Level Emphasis* |
|  | *Zone Percentage* |
|  | *Large Area Low Gray Level Emphasis* |
|  | *Large Area High Gray Level Emphasis* |
|  | *High Gray Level Zone Emphasis* |
|  | *Small Area Emphasis* |
|  | *Low Gray Level Zone Emphasis* |
|  | *Zone Entropy* |
|  | *Small Area Low Gray Level Emphasis* |

Table S2. Association between white matter segmented ROI and radiomics features index.

| ROI | Index |
| --- | --- |
| wm-lh-bankssts | 0 |
| wm-lh-caudalanteriorcingulate | 1 |
| wm-lh-caudalmiddlefrontal | 2 |
| wm-lh-cuneus | 3 |
| wm-lh-entorhinal | 4 |
| wm-lh-fusiform | 5 |
| wm-lh-inferiorparietal | 6 |
| wm-lh-inferiortemporal | 7 |
| wm-lh-isthmuscingulate | 8 |
| wm-lh-lateraloccipital | 9 |
| wm-lh-lateralorbitofrontal | 10 |
| wm-lh-lingual | 11 |
| wm-lh-medialorbitofrontal | 12 |
| wm-lh-middletemporal | 13 |
| wm-lh-parahippocampal | 14 |
| wm-lh-paracentral | 15 |
| wm-lh-parsopercularis | 16 |
| wm-lh-parsorbitalis | 17 |
| wm-lh-parstriangularis | 18 |
| wm-lh-pericalcarine | 19 |
| wm-lh-postcentral | 20 |
| wm-lh-posteriorcingulate | 21 |
| wm-lh-precentral | 22 |
| wm-lh-precuneus | 23 |
| wm-lh-rostralanteriorcingulate | 24 |
| wm-lh-rostralmiddlefrontal | 25 |
| wm-lh-superiorfrontal | 26 |
| wm-lh-superiorparietal | 27 |
| wm-lh-superiortemporal | 28 |
| wm-lh-supramarginal | 29 |
| wm-lh-frontalpole | 30 |
| wm-lh-temporalpole | 31 |
| wm-lh-transversetemporal | 32 |
| wm-lh-insula | 33 |
| wm-rh-bankssts | 34 |
| wm-rh-caudalanteriorcingulate | 35 |
| wm-rh-caudalmiddlefrontal | 36 |
| wm-rh-cuneus | 37 |
| wm-rh-entorhinal | 38 |
| wm-rh-fusiform | 39 |
| wm-rh-inferiorparietal | 40 |
| wm-rh-inferiortemporal | 41 |
| wm-rh-isthmuscingulate | 42 |
| wm-rh-lateraloccipital | 43 |
| wm-rh-lateralorbitofrontal | 44 |
| wm-rh-lingual | 45 |
| wm-rh-medialorbitofrontal | 46 |
| wm-rh-middletemporal | 47 |
| wm-rh-parahippocampal | 48 |
| wm-rh-paracentral | 49 |
| wm-rh-parsopercularis | 50 |
| wm-rh-parsorbitalis | 51 |
| wm-rh-parstriangularis | 52 |
| wm-rh-pericalcarine | 53 |
| wm-rh-postcentral | 54 |
| wm-rh-posteriorcingulate | 55 |
| wm-rh-precentral | 56 |
| wm-rh-precuneus | 57 |
| wm-rh-rostralanteriorcingulate | 58 |
| wm-rh-rostralmiddlefrontal | 59 |
| wm-rh-superiorfrontal | 60 |
| wm-rh-superiorparietal | 61 |
| wm-rh-superiortemporal | 62 |
| wm-rh-supramarginal | 63 |
| wm-rh-frontalpole | 64 |
| wm-rh-temporalpole | 65 |
| wm-rh-transversetemporal | 66 |
| wm-rh-insula | 67 |

Table S3. XGBoost classification performance of clinical/cognitive + volumetry model between groups on test set.

| **Metrics** | **HC vs. svPPA** | **HC vs. nfvPPA** | **svPPA vs. nfvPPA** |
| --- | --- | --- | --- |
| Sensitivity | 1 | 0.778 | 0.889 |
| Specificity | 0.8 | 0.875 | 0.75 |
| Balanced Accuracy | 0.9 | 0.823 | 0.819 |
| F1-score | 0.889 | 0.823 | 0.799 |
| AUC-ROC | 1 | 0.958 | 0.958 |
| Precision | 1 | 0.77 | 0.857 |

HC, Healthy Controls; nfvPPA, non-fluent variant of Primary Progressive Aphasia; svPPA, semantic variant of Primary Progressive Aphasia; AUC-ROC, Area Under the Curve of the Receiver Operating Characteristic.

Table S4. Best hyperparameters for model trained with clinical measures

| **Hyperparameter** | **HC vs. svPPA** | **HC vs. nfvPPA** | **svPPA vs. nfvPPA** |
| --- | --- | --- | --- |
| Learning rate | 0.0407 | 0.0164 | 0.0207 |
| Max depth | 6 | 8 | 7 |
| N° estimators | 142 | 71 | 120 |
| subsample | 0.9982 | 0.6702 | 0.8667 |

Table S5. Best hyperparameters for model trained with clinical and radiomics measures

| **Hyperparameter** | **HC vs. svPPA** | **HC vs. nfvPPA** | **svPPA vs. nfvPPA** |
| --- | --- | --- | --- |
| Learning rate | 0.0302 | 0.1001 | 0.0978 |
| Max depth | 5 | 3 | 4 |
| N° estimators | 90 | 118 | 66 |
| subsample | 0.6618 | 0.7591 | 0.5557 |
